# Complex ventral hernia repair in a child: An association of botulinum toxin, progressive pneumoperitoneum and negative pressure therapy. A case report on an arising surgical technique

**DOI:** 10.1016/j.ijscr.2021.105828

**Published:** 2021-03-23

**Authors:** Marcelo C. Rombaldi, Caroline G. Barreto, Carlos A. Peterson, Leandro Totti Cavazzola, Paola M.B. Santis-Isolan, José Carlos Fraga

**Affiliations:** a Department of Pediatric Surgery, Hospital de Clínicas, Porto Alegre, Brazil; b Department of General Surgery, Hospital de Clínicas, Porto Alegre, Brazil; c Universidade Federal do Rio Grande do Sul, Porto Alegre, Brazil

**Keywords:** GO, giant omphalocele, BTA, botulinum toxin agent, PPP, preoperative progressive pneumoperitoneum, EAF, enteroatmospheric fistula, NPWT, negative pressure wound therapy, PN, parenteral nutrition, pod, pos-operative day, PR, primary early repair, SR, staged early repair, DNR, delayed non-operative repair, PICU, Pediatric Intensive Care Unit, Giant omphalocele, Botulinum toxin, Complex hernia, Enteric fistula, Negative pressure wound therapy, Case report

## Abstract

**Introduction:**

The purpose of this manuscript is to report the management of a child born with giant omphalocele (GO) that developed a complex ventral hernia secondary to an unsuccessful attempt of closing the primary defect.

**Presentation of case:**

The patient underwent a one-step surgery to correct a ventral hernia associated with a largely prolapsed enteroatmospheric fistula (EAF) along with an ileostomy. It was managed by a pre-operative association of botulinum toxin agent (BTA) application with preoperative progressive pneumoperitoneum (PPP) and trans-operative negative pressure wound therapy (NPWT) dressing with staged abdominal closure. The patient needed 4 reoperations due to enteric fistulas. Nine days after the first surgery, it was possible to completely close the abdominal wall without mesh substitution. No signs of hernia in 9 months of follow-up.

**Discussion:**

This is the second report in the literature and it reinforces the safety and effectiveness of the BTA injection associated with PPP in children.

**Conclusion:**

The use of BTA in association with PPP should be encouraged and best investigated in patients with GO. The fistulas were not attributed to the negative pressure. Maybe it is time to start defining better criteria to categorize GO in order to choose the best management for each patient.

## Highlights

• Giant omphalocele establish a therapeutic challenge to the surgeon - mainly because of the increased visceroabdominal disproportion and underlying malformations - and the best approach is still debatable worldwide.
• This is the second report on the literature and states the management of a child born with giant omphalocele that developed a very complex ventral hernia secondary to an unsuccessful attempt of closing the primary defect.
• It seems that the use of botulinum toxin agents in the abdominal wall is safe and effective in children with giant omphaloceles and it eliminates the use of a mesh even in more difficult cases.
• This technique seems safe and effective and it should be encouraged and best evaluated.
• It is time to start defining better criteria to categorize giant omphalocele in order to choose the best management for each patient.

## 1 Introduction

Gian omphaloceles (GO) are most commonly characterized as a defect >5 cm or with a herniated liver [1]. The size of the defect, increased visceroabdominal disproportion, the volume of liver in the sac as well as a high incidence of associated anomalies all together establish a therapeutic challenge [2].

Several centers advocate for primary early repair (PR) or staged early repairs (SR), but the "paint and wait" or delayed non-operative (DNR) strategies has become more frequent, in which the membrane is treated with a topical agent until epithelialization [3]. These patients undergo repair of the hernia later, and several closure techniques are available [4].

Recently, our center reported a combined approach of botulinum toxin agent (BTA) applied to the abdominal wall and preoperative progressive pneumoperitoneum (PPP) in a child with GO [5]. This technique showed to be feasible and safe.

Negative pressure wound therapy (NPWT) has been used in congenital abdominal wall defects and in the management of the open abdomen, complex wounds and enteroatmospheric fistula (EAF) [[6], [7], [8]].

Complex ventral hernias are not common in children and the management of enteric fistulas are also a real struggle to the surgeon.

The purpose of this manuscript is to report the management of a complex ventral hernia using a combination of techniques in a child with giant omphalocele.

All procedures were performed by a pediatric surgery fellow in his fifth year of specialty training and always under senior staff supervision. This article has been written in line with the SCARE criteria [9].

## 2 Presentation of case

**Fig. 1:**
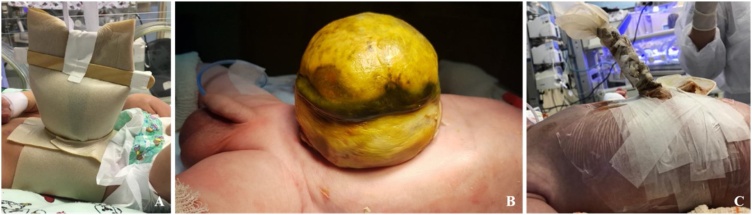
A: Hydrocolloid dressing. B: Omphalocele with bowel perforation and meconial content within the sac. C: Complete content reduction into the abdominal cavity.

A male boy was born in our hospital with a prenatal diagnosis of GO. Patient’s family were young, healthy and stated no use of medication for chronic diseases. No gestational disorders. No history of other genetic/inheritable conditions or malformations reported by the parents. The patient was born with a gestational age of 38 + 4, weighting 3130 g, no underlying malformations. At the neonatal period, he was initially managed with hydrocolloid dressing (Fig. 1a). At 12 days of life, he presented with bowel perforation within the membrane (Fig. 1b). He was taken to surgery and a small colonic perforation was primarily sutured and a silo crafted.

At 22 days of life, he underwent surgical reintervention due to suture dehiscence. A right colectomy with terminal ileostomy was executed. Silo reduction was performed daily until completely reduced after 20 days (Fig. 1c).

**Fig. 2:**
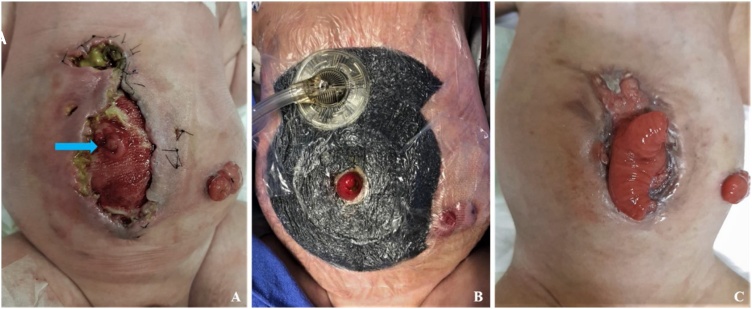
A: Early post-operative eventration and enteroatmospheric fistula (blue arrow). B: Negative pressure wound therapy dressing excluding fistula and ileostomy. C: NPWT result with wound epithelialization and maintained and prolapsed fistula.

At 2 months old an unsuccessful attempt on closing the abdominal wall led to wound infection, dehiscence and EAF (Fig. 2a). It was managed with NPWT dressing (Fig. 2b) until complete wound epithelialization 18 days after, but it didn’t close the EAF (Fig. 2c). He was assessed as having short bowel and was kept admitted until parenteral nutrition (PN) was achieved. He was discharged on enteral and PN, an ileostomy and a ventral hernia with a prolapsed EAF.

**Fig. 3:**
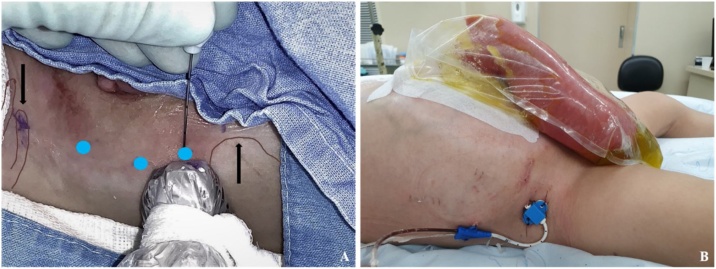
A: BTA application sites ultrasound guided (blue dots) between and 12° rib and anterosuperior iliac spine (black arrows). B: PPP site of catheter insertion.

At 14 months old, due severe prolapse of the fistula as well as short bowel aggravation, it was decided to repair the abdomen in a one-time surgery. Firstly, the patient underwent BTA injection. Under general anesthesia and ultrasound-guided a total of 10U/kg (100U) was applied in six sites (Fig. 3a) of the lateral abdominal wall between the 12th rib and the anterosuperior iliac spine and injected between the transversus and the internal oblique muscles sheets. He was discharged on the same day.

Two weeks after, the patient was submitted to a laparoscopy for abdominal catheter insertion for the PPP. Some adhesions were released and a 7Fr catheter was inserted through direct vision with the tip positioned on the right hypochondrium (Fig. 3b). The patient was kept hospitalized and daily injections of atmospheric air were performed up to maximum tolerance of the patient. No intra-abdominal pressure measure was needed during this period. Approximately 1630 mL was injected in total.

**Fig. 4:**
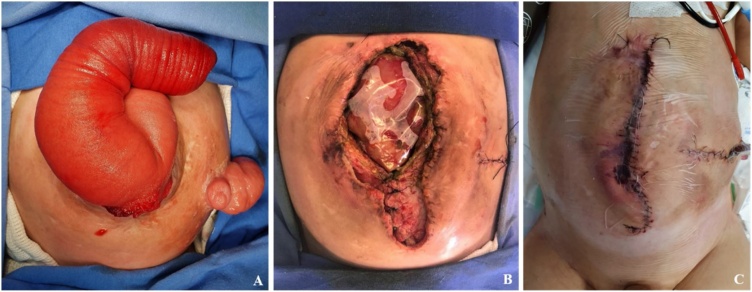
A: Preoperative abdominal condition – ventral hernia with large prolapsed fistula and ileostomy. B: Partial abdominal wall closure. C: Immediate post-operative aspect with “under-skin” NPWT dressing.

Ten days after the PPP, he started with abdominal pain and hemoglobin loss. He was scheduled for fistula correction with abdominal wall reconstruction and ileostomy closure (Fig. 4a).

Laparotomy with adhesiolysis and fifteen centimeters of the prolapsed fistula were resected and a primary anastomosis was performed and the ileostomy was closed. A bleeding with no source was found in the right upper quadrant, related to the tip of the catheter. It was opted for a partial abdominal wall closure as the intra-abdominal and positive end-expiratory pressures were too high. Interrupted 2-0 PDS sutures were used in the bottom half (Fig. 4b) of the defect and the remaining upper border was covered with NPWT dressing (Fig. 4c). The patient was sent to the Pediatric Intensive Care Unit (PICU) and was kept on mechanical ventilation, sedation and curarization, along with total parenteral nutrition (PN) and prophylactic antibiotics for 24 h.

On post-operative day (pod) 3, enteric drainage through the NPWT was observed. He was taken to the operating room and a jejunal perforation was identified and we performed resection of a 2 cm segment and primary anastomosis was performed and the same NPWT dressing was made. On pod 5, he presented with enteric drainage, underwent surgery and suture dehiscence was identified: segmentary resection (3 cm), primary anastomosis and NPWT was placed. Again, on pod 7, due to enteric drainage through the dressing he underwent surgery and a new site of perforation was identified, resected a small segment (1 cm) and primarily anastomosed and a subcutaneous closed suction drain was placed. On pod 9 he had passed stool and was clinically stable. He was taken to the OR and it was decided to completely close the abdominal wall, achieving an immediate intra-abdominal pressure of 13 mmHg, and no repercussion on ventilatory pressures. No mesh was needed. The histopathology for the resected segments reveals a chronic inflammation on the bowel. No fistulogram was performed due to the perforations were clearly secondary to anastomosis dehiscence or previous damage on the intestinal serous an no signal of distal obstruction was observed on the surgery.

Sedation was suspended and he was extubated 24 h after and bowel movement was present on the same day. Enteral nutrition was initiated on pod 6 after complete closure (day 15 after first surgery). On pod 26, he developed a low output enterocutaneous fistula which was successfully managed conservatively with nothing per oral. He was discharged from PICU after complete fistula closure and enteral acceptance, 5 days after.

He was kept hospitalized to optimize enteral PN and enteral intake and was discharged on pod 43 on intermittent home PN.

**Fig. 5:**
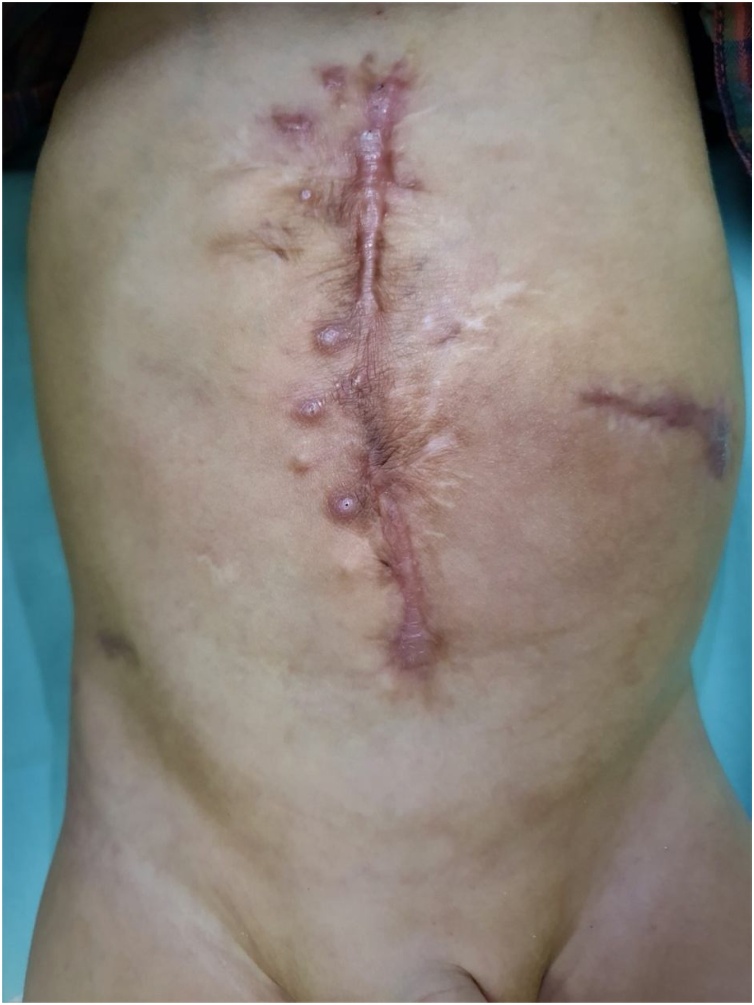
Ninth month post-operative follow up result.

At the twelve-month follow-up showed no signs of hernia, full oral intake and gradually reducing PN, now twice a week (Fig. 5). No other complications were observed.

The patient’s family was extremely satisfied and thankful with the results and all the care provided by the team. During hospitalization, the family was followed by the Psychology department, and even though it was a very stressful period, they were always in accordance and respecting the team’s orientation, mainly when indicating the interventions.

## 3 Discussion

GO management is still arguable worldwide. It seems that 30–50% of all omphaloceles can be closed primarily [2]. Some advocate PR or SR, with the benefits of a shorter length of stay while others advocate DNR for lower infection and mortality rates which may potentially improve long-term neurodevelopment [2,3,[10], [11], [12]].

Nolan [1] reported a 41.5% rate of mesh application on GO and reports show rates of post-operative herniation up to 58% in patients submitted to early closure [4]. Roux [12] showed that the majority of patients underwent DNR and not PR and 96.5% needed a prosthetic substitution. Also, 82.7% of the patients presented any sort of complications, including, infection related to the patch, bowel obstruction, pulmonary dysplasia and death.

Either way, our experience shows that all patients with GO submitted to early repair either developed major complications or are discharged home with a ventral hernia. Thus, in our knowledge the DNR is less aggressive and the patients who tolerate it are discharged home safely with a hernia for a later safer repair.

This case states a GO with massive visceroabdominal disproportion and was initially taken to surgery due to bowel perforation within the membrane. He ended up developing a hostile abdomen as a result of GO complications and a failed attempt to perform an early closure. The extensive adhesiolysis in the first surgery could have played a substantial role in the bowel perforations by possible thermic and serous injuries leading to poor vascularization.

Complex ventral hernias are more often seen in adults than in children.

The combination of BTA and PPP in adults with large ventral hernias has clearly proven its benefits [13,14] but the use of this technique in children was previously described by our center as being the first reported case in the indexed literature [5]. BTA application seems safe in children, but until now, no accurate dose is known for abdominal wall usage [15].

In this particular case, the inefficacy and the complications of the PPP played a key role on the impediment on closing the abdominal wall in the first surgery. Even so, we were able to achieve a great approximation of the median line with just BTA application.

The choice not to ostomize in any of the reoperations was due the good viability of the intestines. This was feasible by the NPWT dressing that aided not only in the early diagnosis of the fistulas but also in keeping the abdomen clear of enteric drainage avoiding peritonitis development. Furthermore, the NPTW aided on maintaining the medial vector strength of the natural lateral vectors of the abdominal wall. In our knowledge the negative pressure had no role on the fistula development.

The success achieved on closing a post-operative fistula by doing resection and primary anastomosis is well defined on the literature [16]. Likewise, the use of the NPWT for complicated abdominal wounds, fistula closure and even congenital wall defects can be safely used in children of any age or weight [[6], [7], [8],^17^].

This is a very complex and challenging case in which was possible to close both the fistula and the ileostomy as well as the abdominal cavity completely without the use of mesh or abdominal wall substitutions.

## 4 Conclusion

The best approach on GO is still not clear. On one side the DNR is less aggressive and the patients who tolerate it are discharged safely with a hernia for a later, safer repair.

It has come to our attention that it is difficult to compare studies in the literature as most of them does not report a rate of mesh application and complications related.

In our knowledge the PPP is essential for dealing with the visceroabdominal disproportion while BTA is more effective for managing the size of the defect. Although debatable, we do not attribute the negative pressure as the cause of the fistulas.

This is the second report in the indexed literature that reinforces the safety and effectiveness of the BTA injection associated with PPP in children. However, we consider that the association of these two techniques should be encouraged and best investigated in patients with GO.

Maybe it is time to start defining better criteria to categorize GO in order to choose the best management for each patient.

## Declaration of Competing Interest

All authors declare that they have no conflict of interest.

## Funding

This research did not receive any specific grant from funding agencies in the public, commercial, or not-for-profit sectors.

## Ethical approval

This is a case report study. Approval from an institutional board review is not required for a case report. Informed patient written consent has been obtained and all identifying information was omitted.

## Consent

Written informed consent was obtained from the patient for publication of this case report and accompanying images. A copy of the written consent is available for review by the Editor-in-Chief of this journal on request.

## Author contribution

**MC Rombaldi:** Case management, conceptualization, article drafting, image editing; **CG Barreto:** Case management, conceptualization, article drafting, image acquisition; **CA Peterson:** Case management, conceptualization, project administration and investigation; **LT Cavazzola:** Case management, investigation, reviewing and editing; **PMB Santis-Isolan:** Final approval, overall supervision **JC Fraga:** Final approval, overall supervision.

## Registration of research studies

Not Applicable.

## Guarantor

Marcelo C Rombaldi; Caroline G Barreto; Carlos A Peterson.

## Provenance and peer review

Not commissioned, externally peer-reviewed.
